# PROGRESS – prospective observational study on hospitalized community acquired pneumonia

**DOI:** 10.1186/s12890-016-0255-8

**Published:** 2016-07-28

**Authors:** Peter Ahnert, Petra Creutz, Markus Scholz, Hartwig Schütte, Christoph Engel, Hamid Hossain, Trinad Chakraborty, Michael Bauer, Michael Kiehntopf, Uwe Völker, Sven Hammerschmidt, Markus Loeffler, Norbert Suttorp, the PROGRESS study group

**Affiliations:** 1 Institute for Medical Informatics, Statistics, and Epidemiology (IMISE), Medical Faculty, University of Leipzig, Haertelstr. 16-18, 04107 Leipzig, Germany; 2 Department of Infectious Disease and Respiratory Medicine, Charité - University Medicine Berlin, Campus Virchowklinikum, Augustenburgerplatz 1, 13353 Berlin, Germany; 3 Department of Pulmonary Medicine, Ernst von Bergmann Hospital, Charlottenstr. 72, 14467 Potsdam, Germany; 4 Institute of Medical Microbiology, Justus-Liebig University Giessen, Schubertstr. 81, 35392 Giessen, Germany; 5 Department of Anesthesiology and Intensive Medicine, Jena University Hospital, Erlanger Allee 101, 07747 Jena, Germany; 6 Integrated Biobank Jena (IBBJ) and Institute of Clinical Chemistry and Laboratory Diagnostics, Jena University Hospital, Erlanger Allee 101, 07747 Jena, Germany; 7 Interfaculty Institute for Genetics and Functional Genomics, Department of Functional Genomics, Ernst-Moritz-Arndt University Greifswald, Friedrich-Ludwig-Jahn-Str. 15a, 17487 Greifswald, Germany; 8 Interfaculty Institute for Genetics and Functional Genomics, Department Genetics of Microorganisms, Ernst-Moritz-Arndt University Greifswald, Friedrich-Ludwig-Jahn-Str. 15a, 17487 Greifswald, Germany; 9 PROGRESS - Pneumonia Research Network on Genetic Resistance and Susceptibility for the Evolution of Severe Sepsis , http://capnetz.de/html/progress/project

**Keywords:** Pneumonia, Sepsis, Prospective observational study, Disease progression, Biomarkers, Innate immunity, Data base, Biobank

## Abstract

**Background:**

Community acquired pneumonia (CAP) is a high incidence disease resulting in about 260,000 hospital admissions per year in Germany, more than myocardial infarction or stroke. Worldwide, CAP is the most frequent infectious disease with high lethality ranging from 1.2 % in those 20–29 years old to over 10 % in patients older than 70 years, even in industrial nations. CAP poses numerous medical challenges, which the PROGRESS (Pneumonia Research Network on Genetic Resistance and Susceptibility for the Evolution of Severe Sepsis) network aims to tackle: Operationalization of disease severity throughout the course of disease, outcome prediction for hospitalized patients and prediction of transitions from uncomplicated CAP to severe CAP, and finally, to CAP with sepsis and organ failure as a life-threatening condition. It is a major aim of PROGRESS to understand and predict patient heterogeneity regarding outcome in the hospital and to develop novel treatment concepts.

**Methods:**

PROGRESS was designed as a clinical, observational, multi-center study of patients with CAP requiring hospitalization. More than 1600 patients selected for low burden of co-morbidities have been enrolled, aiming at a total of 3000. Course of disease, along with therapy, was closely monitored by daily assessments and long-term follow-up. Daily blood samples allow in depth molecular-genetic characterization of patients. We established a well-organized workflow for sample logistics and a comprehensive data management system to collect and manage data from more than 50 study centers in Germany and Austria. Samples are stored in a central biobank and clinical data are stored in a central data base which also integrates all data from molecular assessments.

**Discussion:**

With the PROGRESS study, we established a comprehensive data base of high quality clinical and molecular data allowing investigation of pressing research questions regarding CAP. In-depth molecular characterization will contribute to the discovery of disease mechanisms and establishment of diagnostic and predictive biomarkers. A strength of PROGRESS is the focus on younger patients with low burden of co-morbidities, allowing a more direct look at host biology with less confounding. As a resulting limitation, insights from PROGRESS will require validation in representative patient cohorts to assess clinical utility.

**Trial registration:**

The PROGRESS study was retrospectively registered on May 24^th^, 2016 with ClinicalTrials.gov: NCT02782013

## Background

Community acquired pneumonia (CAP) is truly a high incidence disease (5–10 cases/1000 inhabitants, up to 80 cases/1000 nursing home residents) which results in about 260,000 hospital admissions per year in Germany with an overall mortality for hospitalized CAP of about 13 % [2, 19, 35]. CAP is the most frequent infectious disease worldwide with high lethality [37]. Pneumonia induced severe sepsis frequently jeopardizes successful patient outcome in many medical situations [1]. Severe sepsis and septic shock are the main causes of death on non-coronary intensive care units [1, 4, 7]. According to a recent study by Dremsizov et al., almost half of all patients with severe CAP developed sepsis in the PORT study [6]. In Germany, the SepNet consortium has collected important data on CAP as cause of sepsis in a study of prevalence of severe sepsis and septic shock in German Intensive Care Units (ICUs). In a prospective, observational, cross-sectional study (454 randomly selected ICUs in 310 hospitals in Germany, 3.877 patients screened), respiratory tract infections were identified in 62.9 % of patients with severe sepsis; 39.1 % of infections were considered as community acquired [7]. There is clear evidence that in adults incidence of CAP requiring hospitalization increases with age [2, 9]. Lethality shows a similar pattern: AQUA-data reveal that lethality of hospitalized CAP goes up from about 1.2 % in those 20–29 years old to 12.3 % in the group 70–79 years old and even 22.5 % in the group older than 90 years [2]. In consequence, the health and economic impact of pneumonia is substantial [35].

To date, substantial research efforts over many years have illuminated the origin of CAP, its epidemiology and potential new avenues for treatment [31]. Nevertheless, major challenges remain: While several scoring systems have been developed, their main strength lies in identifying patients not requiring treatment in a hospital [18]. Assessment of disease severity still mainly relies on clinical judgement and outcome prediction remains a challenge [3, 10, 21, 24]. The current lack of a validated operationalization of disease severity throughout the course of treatment in the hospital may hamper clinical research [18].

To meet these challenges and to better understand what determines individual immune responses to pneumonia we conduct a multi-centric observational study of hospitalized patients with CAP in Germany and Austria to establish a comprehensive database of high quality clinical and molecular data. Data obtained will provide the basis for clinical and molecular scoring systems helping clinicians to decide which CAP patient can stay on a regular ward and who is at high risk to progress to severe pneumonia or even septic shock with necessity for intensive care including artificial respiration. The systematic analytical discovery-driven approach chosen will help to identify yet unknown targets and molecular pathomechanisms in the complex dynamics of endothelial barrier function during acute inflammation of the lung [12, 22]. These may directly improve pneumonia management (diagnostics, therapy) and contribute to a better understanding of the pathophysiology of the pneumonia-to-sepsis progression, which in turn will accelerate the identification of new treatment options.

Current research supports the notion that broad as well as in-depth molecular analyses may contribute to a better understanding of disease mechanisms and also lead to diagnostic and prognostic biomarkers [14, 29, 38]. Patients with and without infection may be distinguishable by e.g. gene expression profiles [5, 16, 28, 32]. Even causal pathogens may be detectable by analyzing host biospecimen [25]. A strong association between death from infection in adoptees and their biological, but not adoptive parents suggested a genetic influence on the risk for and outcome from infection [30]. More recently, in the GenOSept study, analysis of data from three cohorts suggested genetic variants associated with survival from sepsis due to pneumonia [26]. It appears worthwhile to study pneumonia systematically and to collect clinical and concomitant molecular data as a basis for further insights.

We here present the design of the PROGRESS study which aims to provide new and in-depth clinical and molecular data helping to elucidate variation in host response to CAP. We hope to contribute to the development of an operationalization of disease severity, to the identification of clinical and biomarkers of pneumonia progression in hospitalized patients, and to new therapeutic concepts. Here, we describe clinical and molecular assessments of the PROGRESS study to facilitate future co-operation and complementary work related to the pressing research questions in CAP.

## Methods/Design

### Study design and assessments

**Fig. 1:**
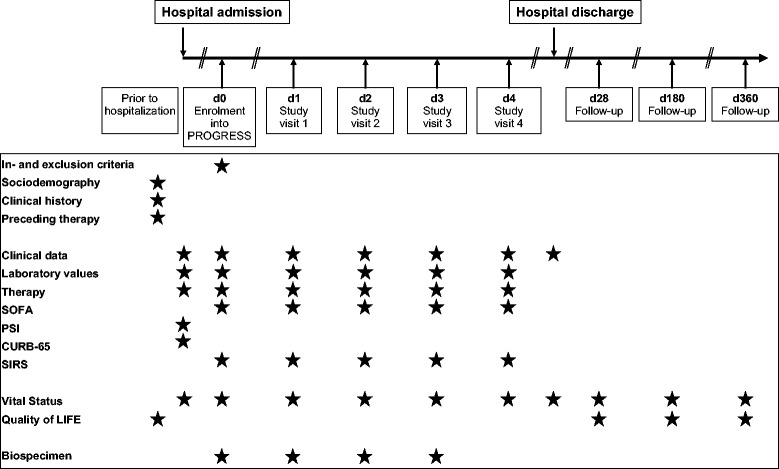
Schematic view of the PROGRESS Study. Stars indicate the time periods or time points from which data originate. Data from study visits correspond to the time range between the previous and current study visit. Patients must be enrolled within 48 h after hospitalization. Study visits correspond to the time points when blood for biospecimen is drawn in the morning. If there would be less than 12 h between collection of biospecimen at enrolment and at visit 1, visit 1 is postponed to the following morning

PROGRESS is a prospective multi-centric longitudinal observational study on patients hospitalized due to confirmed CAP (Fig. 1). Biomaterials are collected for broad molecular investigation. The study does not intend to influence treatment decisions; treatment is at the discretion of the attending physician. PROGRESS is designed to observe transitions from uncomplicated community acquired pneumonia (uCAP) to severe CAP (sCAP) to pneumogenic sepsis and septic shock (ssCAP). Patients are enrolled within 48 h of hospitalization with CAP. Routine clinical and laboratory data are collected for the day of hospitalization, the day of enrolment (d0), and the four following days (d1 through d4). Scores like CURB-65 [20] and PSI [11] are obtained at d0. Parameters for SOFA [33] and SIRS [27] are obtained daily on d0 through d4, along with information on treatment unit, respiratory support, medication, and newly observed clinical and microbiological findings. Blood samples for molecular analyses are collected daily on d0 through d3. At hospital discharge, information like vital status, number of days in intensive care or with respiratory support, and subsequent type of residence are documented. Patients are followed up regarding vital status, quality of life (EQ-5D [15]), and new episodes of pneumonia at days 28, 180, and 360 after enrolment. Baseline data comprise sociodemographic information, relevant aspects of medical history, and treatment prior to hospitalization.

### Study population

PROGRESS aims at recruiting a total of 3000 CAP patients of different degrees of initial disease severity (see inclusion criteria). As PROGRESS aims to provide data on host parameters modulating the response to infection in patients whose immune system appears clinically uncompromised, several exclusion criteria apply (see exclusion criteria). PROGRESS enrolls adult male and female patients from all ethnic backgrounds.

### Inclusion criteria

Study participants must be 18 years of age or older and hospitalized within the past 48 h due to CAP confirmed by a new pulmonary infiltrate on chest radiograph and more than one of the following signs and symptoms of lower respiratory tract infection: 1) Fever, 2) cough, 3) purulent sputum, 4) shortness of breath or need for respiratory support, or 5) crackling or rales on auscultation, dullness to percussion, or bronchial breathing.

### Exclusion criteria

To ensure that the diagnosed pneumonia has not been contracted in a hospital and to avoid patients whose immune system may have been substantially compromised for other reasons, the following exclusion criteria are applied: Patients must not have been hospitalized for any reason within 28 days prior to hospitalization for the current episode of CAP. Patients cannot be enrolled with known HIV infection or AIDS or immunosuppressive treatments within the past six months including anti-tumor treatment, non-steroidal immunosuppressive therapy, radiation therapy, or therapy with corticosteroids ≥ 20 mg for ≥ 14 days. Further exclusion criteria comprise poststenotic pneumonia in conjunction with bronchial carcinoma, organ or bone marrow transplant, respiratory support at home via tracheostoma, cystic fibrosis, active tuberculosis, acute lung injury or acute respiratory distress syndrome for extrapulmonary reasons, massive aspiration, sepsis with extrapulmonary focus, acute pulmonary embolism, congestive heart failure NYHA-IV, or liver insufficiency Child-Pugh C stadium. Also, patients who are pregnant, breast feeding, previously participated in the PROGRESS study, or under limitation of therapy are excluded from the study.

### Study centers

Patients are recruited at study centers throughout Germany and in Austria (Table 1), comprising emergency wards and wards with normal, intermediate, or intensive care. To qualify, a potential study center has to regularly treat CAP patients, have capabilities to process serum and plasma samples using a refrigerated centrifuge and store them at -20 °C, and have access to the internet. To date, more than 50 study centers have contributed over 1600 patients to PROGRESS.

**Table 1:** Sites of Recruitment for the PROGRESS study

PROGRESS Study Site	Location (City, Country, ZIP)	Status
Krankenhaus Angermünde, Klinik f. Innere Medizin/Pneumologie	Angermünde, Germany, 16278	Recruiting
Krankenhaus Bad Arolsen GmbH, Innere Medizin (Herz-, Kreislauf- u. Lungendiagnostik)	Bad Arolsen, Germany, 34454	Recruiting
Hochtaunus-Kliniken, Medizinische Klinik III	Bad Homburg, Germany, 61348	Recruiting
Lungenklinik Ballenstedt/Harz gGmbH, Ev. Fachkrankenhaus f. Lungenkrankheiten	Ballenstedt, Germany, 06493	Recruiting
Vivantes Netzwerk f. Gesundheit GmbH Vivantes Klinikum Neukölln, Klinik f. Innere Med. - Pneumologie u.Infektiologie - Thoraxzentrum	Berlin, Germany, 12351	Recruiting
HELIOS Klinikum Berlin-Buch, Interdisziplinäres Notfallzentrum mit Rettungsstelle	Berlin, Germany, 13125	Recruiting
Charité - Universitätsmedizin Berlin, Medizinische Klinik m. S. Infektiologie und Pneumologie	Berlin, Germany, 13353	Recruiting
Humboldt-Klinikum Vivantes, Kardiologie und kons. Intensivmedizin	Berlin, Germany, 13509	Recruiting
Vivantes Klinikum Spandau, Kard., Pneum. und kons. Intensivmedizin	Berlin, Germany, 13585	Recruiting
Gemeinschaftskrankenhaus Havelhöhe, Kardio-Pneumologie	Berlin, Germany, 14089	Recruiting
Berufsgenossenschaftl. Universitätsklinikum Bergmannsheil GmbH, Klinik f. Pneumologie, Allergologie u. Schlafmedizin	Bochum, Germany, 44789	Recruiting
Evangelische Kliniken Bonn, Betriebsstätte Johanniter Krankenhaus, Innere Medizin II	Bonn, Germany, 53113	Recruiting
Med. Klinik d. Forschungszentrum Borstel, Fachkrankenhaus f. Lungenerkrankungen, Infektionen u. Allergien	Borstel, Germany, 23845	Recruiting
Städt. Klinikum Dessau, Innere Medizin	Dessau-Roßlau, Germany, 06847	Recruiting
Klinikum Dortmund gGmbH, Medizinische Klinik (Pneumologie/Infektiologie)	Dortmund, Germany, 44145	Recruiting
Universitätsklinikum Carl Gustav Carus, TU Dresden, Medizinische Klinik 1 - Abteilung Pneumologie	Dresden, Germany, 01307	Recruiting
SRH Wald-Klinikum Gera gGmbH, Medizinische Klinik 2	Gera, Germany, 07548	Recruiting
LKH-Univ. Klinikum Graz, UKIM Pulmologie	Graz, Austria, 8036	Recruiting
Universitätsklinikum Halle (Saale), Klinik für Innere Medizin III (Kardiologie, Pneumologie, Intensivmedizin, Angiologie)	Halle (Saale), Germany, 06120	Recruiting
Universitätsklinikum Hamburg Eppendorf, Onkologisches Zentrum, Pneumologische Studienzentrale	Hamburg, Germany, 20246	Recruiting
Medizinische Hochschule Hannover, Klinik für Pneumologie	Hannover, Germany, 30652	Recruiting
Klinkum Heidenheim, Medizinische Klinik II & Innere Intensiv	Heidenheim, Germany, 89522	Recruiting
Kliniken d. Main-Taunus-Kreises, Klinik f. Pneumologie u. Allg. Innere Medizin	Hofheim, Germany, 65719	Recruiting
Universitätsklinikum des Saarlandes, Innere Medizin V	Homburg/Saar, Germany, 66421	Recruiting
Universitätsklinikum Jena, Klinik für Anästhesiologie und Intensivtherapie	Jena, Germany, 07747	Recruiting
Universitätsklinikum Jena, Zentrum für Infektionsmedizin und Krankenhaushygiene, Studienabteilung	Jena, Germany, 07747	Recruiting
Evangelisches Krankenhaus Kalk gGmbH, Innere Medizin/Pneumologie	Köln, Germany, 51103	Recruiting
Universität Leipzig, Innere Medizin, Neurologie und Dermatologie, Pneumologie/Studiensekretariat	Leipzig, Germany, 04103	Recruiting
Klinikum St. Georg gGmbH, Klinik für Infektions-/Tropenmedizin und Nephrologie	Leipzig, Germany, 04129	Recruiting
Franziskus Krankenhaus Linz, Innere Abteilung	Linz, Germany, 53545	Recruiting
Universitätsklinikum Schleswig-Holstein - Campus Lübeck, Med. Klinik III (Pneumologie)	Lübeck, Germany, 23538	Recruiting
Universitätsmedizin Mannheim, Studienkoordinierungszentrum, 1. Medizinische Klinik	Mannheim, Germany, 68167	Recruiting
Städtisches Klinikum München GmbH - Klinikum Harlaching, Klinik für Gastroent., Pneum., intern. Akut- u. Intensivmedizin	München, Germany, 81545	Recruiting
Krankenhaus München-Neuperlach, Klinik für Kardiologie, Pneumologie und Internistische Intensivmedizin	München, Germany, 81737	Recruiting
Ev. Fachkrankenhaus für Atemwegserkrankungen	Neustadt, Germany, 99762	Recruiting
Christliches Krankenhaus Quakenbrück e. V., Med. Klinik (Abtl. Pneumologie, Allergologie, Schlafmedizin)	Quakenbrück, Germany, 49610	Recruiting
Mathias-Spital Rheine, Klinik für Pneumologie und Thoraxonkologie	Rheine, Germany, 48431	Recruiting
Diakoniekrankenhaus Rotenburg(Wümme)gGmbH, Zentrum für Pneumologie	Rotenburg/Wümme, Germany, 27356	Recruiting
Altmark-Klinikum gGmbH, Krankenhaus Salzwedel	Salzwedel, Germany, 29410	Recruiting
Krankenhaus Bethanien gGmbH Solingen, Abt. Pneumologie	Solingen, Germany, 42699	Recruiting
Universitätsklinikum Ulm, Klinik für Innere Medizin II, Sudienzentrale Innere II, Sektion Pneumologie	Ulm, Germany, 89081	Recruiting
HELIOS Klinikum Wuppertal, Klinik für Intensivmedizin	Wuppertal, Germany, 42283	Recruiting
HELIOS Klinikum Wuppertal, Pneumologie, Bergisches Lungenzentrum	Wuppertal, Germany, 42283	Recruiting
Missionsärztliche Klinik GmbH Würzburg, Innere Medizin (Pneumologie)	Würzburg, Germany, 97074	Recruiting
Universitätsklinikum Aachen, Med. Klinik I (Pneumologie)	Aachen, Germany, 52057	Completed
Evangel. Krankenhausverein, Luisenhospital, Lungenzentrum	Aachen, Germany, 52064	Completed
Evangelisches Krankenhaus Bad Dürkheim, Innere Medizin	Bad Dürkheim, Germany, 67098	Completed
Knappschaftskrankenhaus Dortmund, Klinik für Pneumologie	Dortmund, Germany, 44309	Completed
Uniklinikum Greifswald, Zentrum für Innere Medizin B	Greifswald, Germany, 17489	Completed
Krankenhaus Martha-Maria Halle-Dölau, Klinik für Innere Medizin I	Halle (Saale), Germany, 06120	Completed
ASKLEPIOS Klinik Wandsbek, 1. Medizinische Abteilung	Hamburg, Germany, 22043	Completed
St.Vincentius-Kliniken gAG, Med. Klinik IV/Pneumologie	Karlsruhe, Germany, 76137	Completed
Allgemeines Krankenhaus der Stadt Linz, Abteilung Lungenheilkunde	Linz, Austria, 4021	Completed
St. Vincenz und Elisabeth Hospital Mainz, Innere Medizin	Mainz, Germany, 55130	Completed
Dietrich-Bonhoeffer-Klinikum, Klinik f. Innere Medizin 2 (Abt. Pulmologie)	Neubrandenburg, Germany, 17039	Completed
Pius-Hospital Oldenburg, Klinik f. Innere Med., Abt. f. Pneumologie	Oldenburg, Germany, 26121	Completed
Brüderkrankenhaus St. Josef, Medizinische Klinik	Paderborn, Germany, 33098	Completed
Universitätsklinikum Regensburg, Klinik Innere Medizin I, Intensivmedizin, Endokrinologie	Regensburg, Germany, 93053	Completed
HELIOS Kliniken Schwerin GmbH, Klinik für Internistische und Neurologische Intensivmedizin/Stroke Unit	Schwerin, Germany, 19049	Completed
Kreisklinik Trostberg, Innere Abteilung	Trostberg, Germany, 83308	Completed

Table 1 lists all study sites which so far contributed patients to the PROGRESS study. Contact to patients can be made through central study management (Petra Creutz: petra.creutz@charité.de or Peter Ahnert: peter.ahnert@imise.uni-leipzig.de).

The study further remains open for participation by interested hospitals. Before initiation of a study center, study personnel have to undergo a comprehensive training program making them familiar with the aims of the study, study procedures, electronic data entry, query management, and sample processing and logistics.

### Data collection and processing

In PROGRESS, study data are collected through standardized web-based data sheets (eCRF) into a central database. PROGRESS aims to collect individual values for all parameters of interest: Scores and derived values are to be calculated centrally to avoid disparity in calculation procedures between study centers, increase opportunities for quality control, allow analysis of individual parameters, and to empower development of new scores and derivatives. Professional trial management and database software are employed for remote data entry and secure storage of clinical data, query management, and basic reporting. Plausibility of data is checked directly upon entry into the eCRF, by several hundred daily queries running on the central database, and in campaigns performed by the data management and biometry teams. Data management actively monitors resolution of identified problems, supported by clinicians from central study management if necessary. Data regarding sample collection, processing, logistics, and from molecular measurements in PROGRESS biospecimen are stored in the same database, ensuring a high level of confidence in sample identity and in matching biospecimen and data from molecular measurements with patient data.

### Ethics and data protection

Written informed consent for participation in the study was obtained from all participants or, in case of critical illness, from patient’s legal representatives. The study protocol has been approved by the ethics committee of the University of Jena (registration number 2403-10/08) and all locally responsible ethics committees of all study centers. The study protocol adheres to the requirements of the Declaration of Helsinki [39] and to the ICH-GCP guideline [8].

To achieve the objective of the study, it is necessary to collect and process data and biospecimen of individual patients. Identification of patients is possible only in the study centers via a study specific patient pseudonym. All inquiries of data management to the study centers as part of quality assurance processes are using the pseudonym. Collected biospecimen are identified by a ten-digit code which is linked to the patient pseudonym at the central database. Identification of specific patients is not required at any time outside the study centers. To ensure that DNA samples cannot be linked to patient identity by anyone involved in the study and to also ensure that DNA samples can be destroyed at patient’s request, information linking DNA samples to patient identity are held by an external data trustee.

### Sample collection and biobanking

Specimen to be collected daily (days d0 through d3) include serum, EDTA plasma with protease inhibitors, citrate plasma, and PAXgene blood for RNA. Samples are collected, processed, and stored at -20 °C for up to three months in the study centers. Samples are then shipped to the Integrated Biobank Jena (IBBJ), the central biobank of the PROGRESS network. A main goal was to minimize the number of pre-analytical steps in study centers to limit heterogeneity introduced by slight differences in personnel and procedures. Centralized and automated sample handling and processing helps to assure standardization and to minimize sample loss and mix-up, greatly contributing to high standards of quality. At the biobank, sample aliquots are kept at -80 °C in a fully automated sample store. Two vials of EDTA whole blood for extraction of DNA are obtained from each patient and shipped directly to the biobank at ambient temperature, then stored at -80 °C until processing.

### Molecular assessments

Broad molecular analyses are an important focus of PROGRESS. All study participants will be genotyped using the CAP2 array, a customized array based on the Axiom platform (Affymetrix). The CAP2 array comprises the standard genome-wide content of the Axiom CEU array and about 60,000 custom SNPs. This custom content consists of SNPs selected from existing literature evidence, eQTL data bases, GWAS data bases, and data bases of functionally relevant mutations. Genetic data from the CAP2 array are suitable for imputation of genotype data with common genomic references. Genome-wide gene-expression measurements using the HumanHT-12v4 Expression BeadChip (Illumina) comprise 47.000 transcript features in RNA from stabilized whole blood (PAXgene Blood RNA System, PreAnalytiX). Proteome analyses involve gel-free LC-MS/MS focused measurements in EDTA plasma samples stabilized by protease inhibitors (BD P100 Blood Collection System for plasma protein preservation, Becton Dickinson) and depleted for highly abundant proteins. Collected biospecimen also allow measurements of other serum and plasma parameters as well as cytokines. As patient samples are available from consecutive study time points, time series data can be established which are amenable to temporal analysis of molecular response to infection.

## Discussion

PROGRESS collects clinical and molecular data for investigating the role of host factors modulating innate immunity in patient’s response to community acquired pneumonia. Most patients hospitalized with CAP recover rather quickly while others will experience a severe course of disease, with some requiring ICU treatment including respiratory support. PROGRESS is designed to investigate host factors beyond old age and comorbidity which modulate the course of disease. Therefore, PROGRESS was designed as a unique, detailed, systematic, longitudinal study of CAP in Germany and Austria focusing on the progression of hospitalized community acquired pneumonia from mild CAP to very severe disease including severe sepsis and septic shock. Patients are closely followed for several days regarding clinical and laboratory parameters and several biospecimen are collected on these days. Inspiration for the design of PROGRESS came from CAPNETZ and its study on epidemiological and health research aspects of CAP in Germany [34, 36]. Main differences of PROGRESS compared to CAPNETZ are very stringent exclusion criteria excluding severe comorbidities, a specific focus on hospitalized CAP, more comprehensive documentation on disease course within the first five days in the hospital, and a stronger focus on molecular assessments. Another inspiration for the PROGRESS study was the “study on Genetic and Inflammatory Markers of Sepsis” (GenIMS) in CAP in the US [17]. However, criteria in PROGRESS are much stricter regarding exclusion of potentially immunocompromised patients and options for molecular assessments are broader. Inspiration regarding the assessment of severely ill CAP patients came from several SepNet [23] studies in Germany which, like the European GenOSept study [13, 26], focus on patients with sepsis of diverse origins, including pneumonia.

Stringent inclusion criteria ensure that only patients with community acquired pneumonia are enrolled. A new infiltrate in chest X-ray along with at least two other clinical symptoms of pneumonia are required. Patients identified at later time points, or suffering from tuberculosis or neoplasia of the lung, for instance, are excluded from the study. To further ensure disease homogeneity, patients who may have acquired pneumonia in medical care facilities (hospital or health care acquired pneumonia, HAP) or under respiratory support (ventilator acquired pneumonia, VAP) are excluded. Numerous exclusion criteria apply in an attempt to avoid patients whose immune systems may be substantially compromised by diseases with direct impact on the immune system, other severe diseases likely to modulate the course of pneumonia, or immunosuppressive treatments. Along these lines, patients for whom a therapy limitation is assumed are excluded. Decisions on limitation of therapy that are made after enrolment are documented. Hence, patients enrolled in PROGRESS are not representative of the overall population of hospitalized CAP patients. Rather, PROGRESS patients will likely be younger and generally healthier except for their acute pneumonia. While our approach aims to get closer to unconfounded biological heterogeneity regarding CAP progression, this also requires that insights from PROGRESS have to be validated in more representative patient cohorts before generalizations regarding all CAP patients can be drawn.

By definition, it is impossible to observe patients hospitalized with CAP without the influence of the hospital environment. To come as close as possible to an analysis of pure CAP, we require enrolment of patients within 48 h of hospitalization and are limiting detailed observation of patients to the first five to six days after arrival in the hospital. At later time points, it cannot be ruled out that hospital acquired infection may superimpose on the initial CAP. However, major events and substantial changes in patients’ health state are recorded until hospital discharge to gain insight into the overall course of disease in the hospital. Patients are followed up by telephone interview at days 28, 180, and 360 after enrolment for vital status, recurrence of pneumonia, housing situation, and quality of life. Thus, questions regarding long term consequences of CAP can also be addressed.

In the PROGRESS study, microbiological data are documented as they become available from routine clinical materials, especially if materials were obtained between hospital admission and day 4 of the study. This includes culture from airway materials and blood as a well as influenza rapid test and urine antigen tests for legionella viruses and pneumococci. Results from molecular techniques such as PCR are also documented. Microbiological assessment will likely be available for only a part of PROGRESS patients and disease causing agents will be detected for only a fraction of patients. We hope to supplement this information on disease causing agents in the long run by molecular analyses of available biospecimen. These analyses can be validated with documented microbiological data.

The main goal of PROGRESS is to contribute to the improvement of clinical decision making and management of patients with pneumonia and sepsis and of therapeutic strategies. This will be achieved by clinical and molecular investigation of host factors modulating outcome of CAP and by identifying clinical scores and molecular signatures for diagnosis, risk stratification, prognosis, and outcome prediction.

## Abbreviations

AIDS, acquired immunodeficiency syndrome; AQUA, Institute for Applied Quality Improvement and Research in Health Care, Germany; CAP, community acquired pneumonia; DNA, deoxyribonucleic acid; eCRF, electronic case report form; EDTA, ethylenediaminetetraacetic acid; eQTL, expression quantitative trait locus; GenIMS, genetic and inflammatory markers of sepsis study; GWAS, Genome Wide Association Study; HAP, Hospital or Health care Acquired Pneumonia; HIV, human immunodeficiency virus; IBBJ, integrated biobank jena; ICU, intensive care unit; LC-MS/MS, liquid chromatography coupled tandem mass spectroscopy; PCR, polymerase chain reaction; PROGRESS, Pneumonia Research Network on Genetic Resistance and Susceptibility for the Evolution of Severe Sepsis; RNA, ribonucleic acid; sCAP, severe CAP; SepNet, Study Group Competency Network Sepsis; ssCAP, severe CAP with pneumogenic sepsis and septic shock; uCAP, uncomplicated CAP; VAP, ventilator acquired pneumonia; WHO, World Health Organization
